# The association between community-level socioeconomic status and depressive symptoms among middle-aged and older adults in China

**DOI:** 10.1186/s12888-022-03937-9

**Published:** 2022-04-28

**Authors:** Yan Liu, Zhaorui Liu, Richard Liang, Yanan Luo

**Affiliations:** 1 Institute of Population Research, Peking University, Beijing, China; 2 Peking University Sixth Hospital, Beijing, China; 3 School of Medicine, Stanford University, Palo Alto, California USA; 4 Department of Global Health, School of Public Health, Peking University, No.38 Xueyuan Road, Haidian District, 100191 Beijing, China

**Keywords:** Chinese middle-aged and older people, Community factors, Depressive symptoms, Socioeconomic status

## Abstract

**Background:**

There was little evidence concerning the association of community socioeconomic status (SES) and the cross-level interaction between community- and individual-level SES with depressive symptoms in China. This study aimed to investigate the association of community-level SES with depressive symptoms among Chinese middle-aged and older people and to examine whether individual-level SES moderates this relationship.

**Methods:**

Using data from the China Health and Retirement Longitudinal 2011–2018 Study, the 10-item Center for Epidemiologic Studies Depression Scale (CES-D-10) short form was used to measure depressive symptoms in 35,546 Chinese individuals aged 45 years and older. Community SES was calculated as a sum of z scores of the average years of schooling and household income per capita, which were derived by aggregating the individual measures to the community level. Two-level hierarchical linear regression was used.

**Results:**

Community SES was negatively related to CES-D-10 scores (coef=-0.438). A 1-SD increase in individual SES was associated with lower CES-D-10 scores (coef=-0.490). The cross-level interaction on individual- and community-level SES was significantly associated with depressive symptoms, indicating that with the increase of individual-level SES, the effect of community-level SES on depression decreases. Stratified analyses observed robust associations of community SES with CES-D scores between urban and rural residents.

**Conclusions:**

This study showed that individuals who live in lower-SES communities had more severe depressive symptoms, particularly individuals with low SES. Additional attention should be given to the community socioeconomic context of middle-aged and older adults with lower SES, which may be helpful to reduce SES inequalities in depressive symptoms in China.

**Supplementary information:**

The online version contains supplementary material available at 10.1186/s12888-022-03937-9.

## Introduction

Depression is a global public health concern, imposing a high-level disease burden on individuals, families and societies [1]. Major depression is predicted to be the first cause of the burden of diseases worldwide by 2030 [2]. As one of the leading causes of disability-adjusted life-years (DALYs) in 2017 [3], depressive disorders can cause psychosocial health problems, chronic illnesses, low quality of life, high risk of suicide and increased utilization of healthcare services [4–6]. It is noteworthy that depression is one of the most predominant mental disorders in middle-aged and older people [6, 7]. The prevalence of depression was reported to peak at approximately 50–60 years old [2]. Based on a nationally representative survey in China, the lifetime prevalence and 12-month prevalence of depressive disorders among adults was 6.8% and 3.6%, respectively, and the 12-month depressive disorder prevalence was 4.1% for people aged 50–64 and 3.8% for older adults aged 65+ [8].

Socioeconomic status (SES) has been found to be associated with a higher risk of depressive symptoms [6, 9, 10]. Although most of them focused on the association between individual-level SES and health, an independent effect of area SES on health has been confirmed by robust evidence [11–18], and even a greater impact of area SES than individual SES on mental health was found [19]. Increased inflammation, environmental exposures (e.g., violence and crimes, toxins, and street connectivity), social capital (e.g., social cohesion, social support and civic participation), the availability of public services (e.g., policing, sanitation and medical care), and access to behavioral resources (e.g., healthy food environments and exercise facilities) may contribute to individual health outcomes as a function of area-level SES [11, 15, 20–22]. Two theories from the neighborhood stress process model and the person-environment perspective also indicated that depressive symptoms were affected by stressors (sources of stress) from low SES at the individual level and the neighborhood level [23–25].

A large number of studies have highlighted the importance of SES for health; however, in comparison to individual SES, the role of community SES has only recently been given the attention it deserves. And whether the relationship between area SES and health will be stronger or weaker depending on individual SES conflicts. The local social inequality model and the relative deprivation hypothesis indicate that the health of residents with lower SES is more likely to be better in lower SES areas than in higher SES areas [11, 14, 20, 26]. Whereas the collective resources theory and the fundamental cause model assume that the health of lower SES individuals is more subject to the socioeconomic status of areas they reside in [11, 14, 20, 26]. Specifically, the double jeopardy hypothesis suggests that the health of individuals with lower SES living in disadvantaged areas is worse than that of those living in advantaged areas [11, 14, 20].

To date, the majority of these studies concerning the association between community-level or neighborhood-level SES and depressive symptoms were from developed countries [10, 26–31]. To our knowledge, only one study in China, using an investigation in urban neighborhoods of Shanghai, has examined neighborhood-level SES and depressive symptoms and found that older adults aged 60 or above residing in lower-SES neighborhoods were related to an increased risk of depression [32]. However, this study did not evaluate the interaction effect of community- and individual-level SES on depressive symptoms.

Using a large-scale nationally representative longitudinal dataset, this study aimed to investigate the association of community-level SES and depressive symptoms among Chinese middle-aged and older people and to examine whether individual-level SES moderates this relationship. In addition, given the considerable socioeconomic disparities between urban and rural communities, we further explored whether these associations vary by urban/rural residence. Our study contributes to the world literature on this issue in the context of developing countries, and it would be of great benefit to identifying the priority populations and communities for the possible prevention of depressive symptoms, which may offer a sound rationale for improving the mental healthcare system in China.

## Methods

### Data and sample

Four waves (2011–2018) of the China Health and Retirement Longitudinal Survey (CHARLS) were used in our study. CHARLS is a nationally representative longitudinal survey among Chinese community-dwelling people aged 45 or older [33]. The survey chose the community to be the primary sampling units (PSUs), which was referred to as rural villages (cun) or urban neighborhoods (shequ). Then, CHARLS adopted a multistage, stratified, probability proportional to size (PPS) random sampling method to extract 450 PSUs from 150 counties/districts across 28 provinces of China. All data were collected by face-to-face computer-aided personal interviews (CAPIs). The first wave (W1) interviewed 17,708 individuals in 2011–2012 with three follow-ups conducted every two years from 2013 to 2018. During 2013–2014, the second wave (W2) reinterviewed 15,186 individuals, among which 13,565 individuals were followed-up in the third wave (W3) 2015–2016, and 11,988 respondents were finally reinterviewed in the fourth wave (W4) 2018–2019. A detailed household questionnaire was used in CHARLS to collect information on demographic characteristics, family, health status, health care, etc. Additionally, CHARLS formulated a separate community-level questionnaire, including information on important infrastructure and health facilities available in the community, the availability of social policy and health welfare, and some basic information of the community (such as the population scale and the history of the community). More detailed information and data on CHARLS can be found at http://charls.pku.edu.cn/en.

**Fig. 1:**
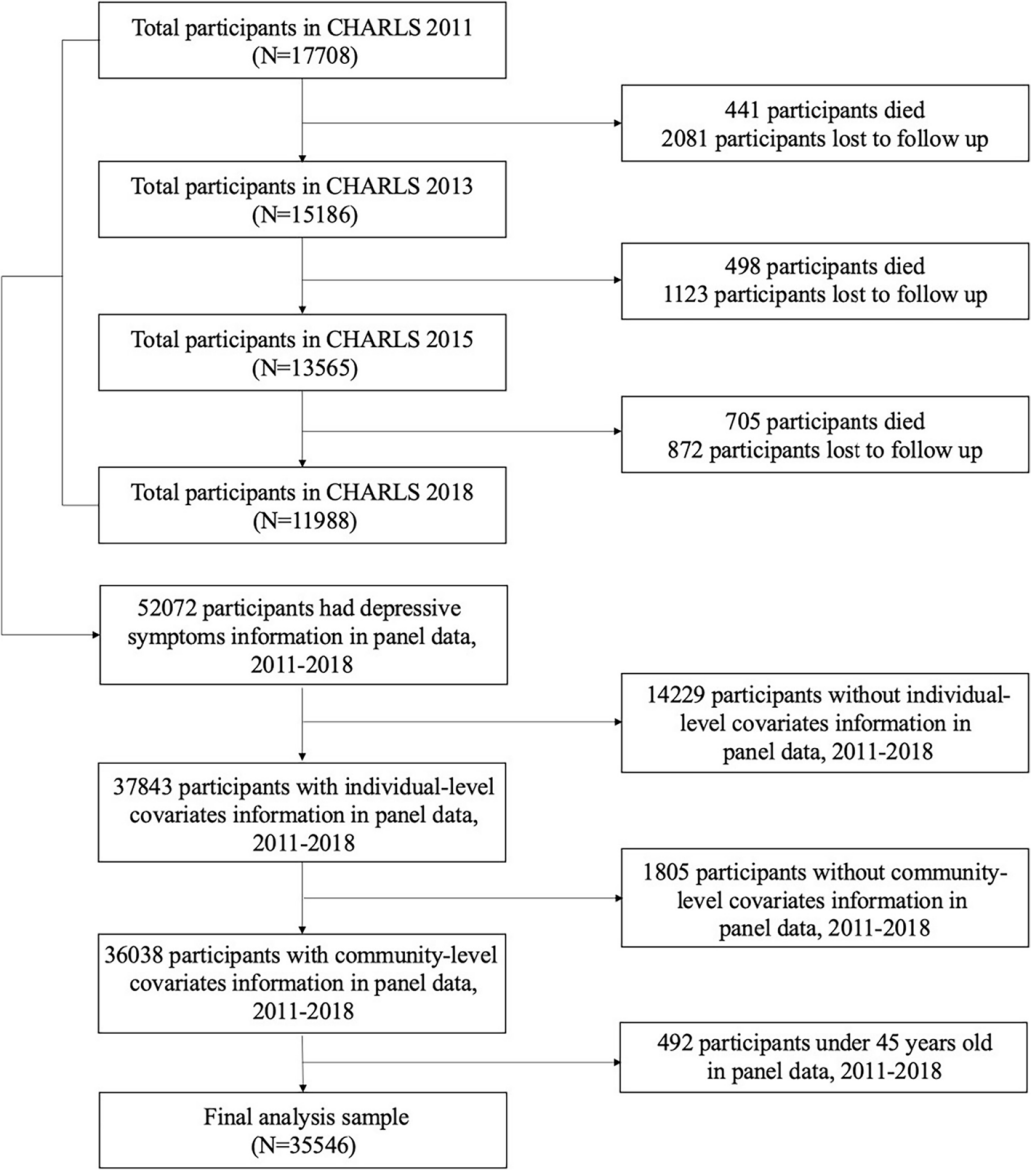
Flow chart of sampling of this study. Notes: CHARLS, China Health and Retirement Longitudinal Survey

Individuals who did not enroll in W2 (2522), W3 (1621) and W4 (1577) were excluded. Then, we excluded 6375 cases without depressive symptom information, participants (14,229) without individual-level covariates and cases (1805) with missing community-level covariate information in waves 2011–2018. Participants younger than 45 years old were excluded, resulting in a final analytical sample of 35,546 observations in 2011–2018. Figure 1 presents a flow chart of the 2011–2018 CHARLS study.

### Measures

#### Depressive symptoms

The 10-item short form of the Center for Epidemiologic Studies Depression scale (CES-D-10) was utilized as a measure of depressive symptoms [34, 35]. The Cronbach’s alpha varied from 0.79 to 0.81, indicating that it is a scale with good validity and reliability in measuring depressive symptoms among Chinese middle-aged and older adults [24, 36, 37]. Respondents were asked to rate the frequency of their mood and behavioral symptoms during the prior week by using the CES-D-10 with answers ranging from ‘rarely or none of the time (less than one day)’ to ‘most or all the time (5–7 days)’. The range of the CES-D-10 score is 0–30, and a higher score means severe depressive symptoms.

#### Community-level SES

Objective community-level information is not publicly available due to the data limitations of the Chinese census. Thus, according to previous studies, we derived community SES indicators by aggregating the individual-level SES measures to the community level [10, 15, 19, 29, 38–41]. In our study, community-level SES was calculated by the sum of z scores of the average years of schooling and household income per capita. Of them, the average years of schooling was obtained from education degree attainment (illiterate/semiliterate = 0, sishu/elementary school = 6, middle school = 9, high school/vocational school = 12, college = 16, postgraduate = 19). Community-level SES had a mean of zero and SD of 1, ranging from − 3.96 to 5.74, with higher values representing higher community SES.

#### Individual-level SES

Corresponding to community-level SES, individual SES was also measured by years of schooling and household income per capita. Similarly, years of schooling was obtained from the categorical variable educational attainment, which was also treated as a continuous variable (illiterate/semiliterate = 0, sishu/elementary school = 6, middle school = 9, high school/vocational school = 12, college = 16, postgraduate = 19). Z scores were calculated for each variable, and their sum was defined as individual-level SES [13, 14]. The range of individual-level SES was from − 0.92 to 38.13 with a mean of zero and SD of 1. Higher values of the indicator indicated a higher level of individual SES.

#### Community-level covariates

At the community level, the physical and social environments of communities were investigated in our study, as both of them have been found to be key determinants of older adults’ health [24, 42–44]. Transportation and outdoor space and buildings were used to measure the community physical environment following previous research [24, 42]. Of these, transportation was measured by the distance to bus stops that people most commonly used (0-100 km). Outdoor space and buildings were measured by the number of days that the roads were not passable in the previous year (0-356 days) and the degree of handicapped access for community dwellers (ranging from no handicapped access = 1 to very convenient = 7). Community support was used to measure the community social environment according to prior studies [42, 44]. Community support was measured by whether the community had the employment service and provided pensions to people aged 65 and older.

#### Individual-level covariates

At the individual level, control variables comprised age in years, sex (female/male), occupation (nonagricultural work/agricultural work), marital status (married/unmarried), residence (rural/urban), and activities of daily living (ADLs) (with impairment/without impairment), which were obtained from a self-reported questionnaire. Individuals with impaired ADLs were defined as those having difficulties in at least one of the ADL items including bathing, dressing, eating, getting in/out of bed, using the toilet, and controlling urination.

### Statistical analysis

Descriptive statistics were conducted to present community-level and individual-level characteristics and depressive symptom status according to various community- and individual-level SES. Two-level hierarchical linear regression models (HLMs) were undertaken to examine the independent relationships between community SES, individual SES, and their interaction and depressive symptoms. Three models were fitted, including the first model allowing for community-level SES and multiple demographic and socioeconomic covariates, the second model further adjusting for individual SES, and the third model adding the interaction term between community-level SES and individual-level SES. The interclass correlation coefficient (ICC) was provided for each model to represent the proportion of variance in depressive symptoms that could be explained by community-level social factors.

In our analytical sample, approximately 11% of participants due to missing data on depressive symptoms, 20% on individual income, and 5% on ADLs were excluded. To address the potential bias caused by sample exclusion, we conducted the analysis using multiple imputation methods by chained equations to replace missing values with five completed data sets [45]. A *p*-value < 0.05 was considered statistically significant. All statistical analyses were performed using Stata V.14.0 (StataCorp LP, College Station, Texas).

## Results

### Characteristics of participants

Table 1 reports the characteristics of the participants from baseline CHARLS 2011. Of all individuals, the mean score of CES-D-10 was 8.51. The average age of the individuals was 59.23 years old in this study. More females (52.14%) and more residents with nonagricultural work (52.58%) were among our participants. Most of the participants were married (86.89%) and had unimpaired ADLs (82.63). At the community level, the days on which roads were unpassable last year had an average of 33.54. The average distance to the most commonly used bus stops was 2.96 km, and the level of handicapped access averaged 1.99. The proportion of employment service and income subsidies for old age was only 20.69% and 22.64%, respectively (see Additional file 1).

**Table 1:** Characteristics of participants from baseline CHARLS 2011

Characteristics	Total (*n* = 12,260)	Urban (*n* = 4821)	Rural (*n* = 7439)
	N (%) or Mean (SD)	N (%) or Mean (SD)	N (%) or Mean (SD)
**Outcome**			
CES-D-10	8.51(6.40)	7.40(6.00)	9.23(6.55)
**Community-level SES variables**			
Average years of schooling, years	4.71(2.22)	6.16(2.39)	3.78(1.49)
Per household income, yuan	9489.10(8739.59)	14576.68(11255.49)	6191.98(4017.76)
Community-level SES	0.00(1.00)	0.70(1.18)	-0.45(0.48)
Community-level SES, n (%) ^a^			
Bottom tertile	4066(33.16)	554(11.49)	3512(47.21)
Middle tertile	4079(33.27)	1078(22.36)	3001(40.34)
Top tertile	4115(33.56)	3189(66.15)	926(12.45)
**Individual-level SES variables**			
Years of schooling, years	4.71(4.68)	6.16(4.93)	3.78(4.26)
Per household income, yuan	9489.10(23034.32)	14576.68(33699.14)	6191.98(10531.57)
Individual-level SES	0.00(1.00)	0.39(1.44)	-0.23(0.74)
Individual-level SES, n (%) ^a^			
Bottom tertile	4079(33.27)	963(19.98)	3116(41.89)
Middle tertile	4094(33.39)	1459(30.26)	2635(35.42)
Top tertile	4087(33.34)	2399(49.76)	1688(22.69)

*CES-D-10 *10-item short form of the Center for Epidemiologic Studies Depression scale, *SES *socioeconomic status

^a^Variable was treated as a continuous variable but is presented categorically for descriptive purposes

In terms of individual-level SES indicators, the average years of schooling and average per household income was 4.71 and 9489.10 yuan, respectively. Concerning community SES indicators, the average years of schooling was also 4.71, and per household income had an average of 9489.10 yuan.

Compared with rural respondents, urban respondents were more likely to have higher individual SES and lower mean CES-D-10 scores and were more possibly to reside in higher SES communities. More details could be found in Table 1.

### Depressive symptoms of participants by SES

Table 2 features the depressive symptoms of samples by different levels of SES from baseline CHARLS 2011. Among all respondents, the mean score for CES-D-10 decreased with the increased individual SES, and the CES-D-10 mean score was 10.59, 8.52 and 6.43 for individuals in the bottom tertile, middle tertile and top tertile, respectively. At the community level, it also found that the mean score for CES-D-10 decreased with the increased community SES. Among both urban and rural residents, the pattern of depressive symptoms was similar to that among all participants by individual-level and community-level SES.

**Table 2:** Depressive symptoms of participants by community-level SES and individual-level SES from baseline CHARLS 2011

Characteristics	Total (*n* = 12,260)	Urban (*n* = 4821)	Rural (*n* = 7439)
	Mean (SD)	Mean (SD)	Mean (SD)
**Community-level SES**			
Bottom tertile	9.88(6.60)	9.69(6.42)	9.91(6.63)
Middle tertile	8.80(6.47)	8.37(6.36)	8.96(6.51)
Top tertile	6.87(5.74)	6.68(5.65)	7.51(6.02)
**Individual-level SES**			
Bottom tertile	10.59(6.78)	10.49(6.64)	10.62(6.82)
Middle tertile	8.52(6.24)	7.89(5.93)	8.87(6.37)
Top tertile	6.43(5.44)	5.87(5.19)	7.22(5.67)
**Community-level SES (= bottom tertile)**			
Individual-level SES			
Bottom tertile	10.75(6.78)	11.05(6.22)	10.71(6.86)
Middle tertile	9.40(6.42)	8.95(6.67)	9.48(6.37)
Top tertile	7.39(5.42)	6.22(4.77)	7.57(5.50)
**Community-level SES (= middle tertile)**			
Individual-level SES			
Bottom tertile	10.49(6.88)	10.23(7.13)	10.58(6.79)
Middle tertile	8.50(6.30)	8.15(6.00)	8.62(6.40)
Top tertile	7.28(5.74)	6.69(5.34)	7.51(5.88)
**Community-level SES (= top tertile)**			
Individual-level SES			
Bottom tertile	10.12(6.48)	10.25(6.45)	9.88(6.56)
Middle tertile	7.61(5.81)	7.54(5.70)	7.78(6.10)
Top tertile	5.80(5.20)	5.73(5.17)	6.18(5.31)

*SES *socioeconomic status

### Community-level SES and depressive symptoms

Table 3 illustrates the results of multilevel linear regressions of the association between community-level SES, individual-level SES, and their interaction and depressive symptoms. Four assumptions of linear relationship, independence, homoscedasticity, and normality for our models were met after the tests for autocorrelation, multicollinearity, homoscedasticity and normality (see Additional file 2). Model 1 confirmed the descriptive analysis of the mean CES-D-10 score in various community SES areas, with depressive symptoms decreasing with community-level SES (coef=-0.438). Model 2 found a slight impact of community-level SES after allowing for individual-level SES (community-level SES: coef=-0.211; individual-level SES: coef=-0.490). Model 3 showed that the interaction term was significantly associated with depressive symptoms (coef = 0.126), which indicates that with the increase of individual-level SES, the effect of community-level SES on depression decreases.

**Table 3:** Multilevel linear regressions of the association between community-level SES, individual-level SES, and their interaction and depressive symptoms, adjusting for covariates

Characteristics	Total			Urban			Rural		
	Model 1	Model 2	Model 3	Model 1a	Model 2a	Model 3a	Model 1b	Model 2b	Model 3b
Community-level SES	-0.438***	-0.211***	-0.223***	-0.434***	-0.226***	-0.286***	-0.437***	-0.202*	-0.166
Individual-level SES		-0.490***	-0.612***		-0.427***	-0.742***		-0.539***	-0.549***
Community-level SES x Individual-level SES			0.126***			0.160***			0.141***
ICC	0.047	0.047	0.047	0.042	0.043	0.042	0.050	0.050	0.050
*R* ^2^									
Individual-level *R*^2^	0.149	0.153	0.154	0.124	0.128	0.132	0.136	0.140	0.140
Community-level *R*^2^	0.109	0.113	0.114	0.096	0.101	0.104	0.114	0.119	0.119

*SES* socioeconomic status, *ICC *interclass correlation coefficient

Model 1: adjusted for community-level sociodemographic variables (distance to bus stop, days roads unpassable, handicapped access, employment service, old-age income subsidies) and individual-level sociodemographic variables (age, sex, residence, occupation, marital status, ADLs)

Model 2: adjusted for Model 1 criteria and individual-level SES

Model 3: adjusted for Model 2 criteria and the interaction between community-level and individual-level SES

*P < 0.05, ***P < 0.001

Furthermore, urban-rural differences in the association between community-level SES and depressive symptoms were not found in the stratified analyses of urban and rural residents. Among those in urban or rural communities, a similar pattern was found to that among the total population.

## Discussion

The aim of this study was to examine the association of community-level SES and its interaction with individual-level SES with depressive symptoms and the urban-rural differences in these association among Chinese middle-aged and older people. Our results suggested that community-level SES was inversely associated with depressive symptoms, namely, individuals who live in higher-SES communities had lower depressive symptom scores. Moreover, the interaction between community-level SES and individual-level SES was significant in predicting depressive symptoms. Stratified analyses also observed that these associations between community SES and CES-D scores were robust in both urban and rural resident groups.

Our study indicated that individuals living in higher SES communities experienced fewer depressive symptoms than their counterparts residing in lower SES communities among Chinese middle-aged and older adults. This result is in line with previous studies in the setting of the UK [26], the United States [28] and the Netherlands [27]. The study from the UK, measuring depression with the General Health Questionnaire (GHQ-30), showed that living in more deprived neighborhoods was related to an increased risk of depression (OR = 1.14, 95% CI: 1.04, 1.24) [26]. Evidence from the United States found that neighborhood SES was protective against worsening depression scores among people aged 50 years or older (coef=-0.48, 95% CI: -0.83, -0.12) by using the assessment of the Patient Health Questionnaire (PHQ-9) for depression [28]. The Netherlands research suggested that neighborhood SES scores were associated with depressive disorders (OR = 0.88, 95% CI: 0.79, 0.98) and the severity of depressive symptoms (coef=-0.06, 95% CI: -0.10, -0.02), with the semistructured Composite International Diagnostic Interview and the Inventory of Depression Symptomatology (IDS-28) as the measure of depressive disorders and its severity, respectively [27].

Several mechanisms may explain the negative relationship between community-level SES and depressive symptoms. For instance, the collective resources model proposes that all individuals, regardless of their SES, are expected to benefit from area-level resources in more advantaged areas, and individuals residing in deprived communities have fewer resources to gain health knowledge and worse availability and accessibility of health care services [12, 15, 20, 26]. Moreover, residents in lower-SES communities may perceive a lower level of social cohesion and are more likely to feel abandoned and forgotten by others surrounding them and the government, which may increase the likelihood of being depressed [15, 35, 46]. Furthermore, residents living in deprived communities may be in community environments with less green space and smaller amounts of sidewalks, so they have a higher risk of depressive symptoms [15].

A striking finding in this study was a significant interplay relationship between individual and community SES with depressive symptoms, indicating that with the increase of individual-level SES, the effect of community-level SES on depressive symptoms decreases. The fundamental cause theory suggests that the positive association between area SES and health was larger among lower-SES individuals than their higher-SES counterparts. Individuals with lower SES tend to be unable to access health-enhancing resources privately and be more dependent on services provided by the local communities, whereas higher-SES individuals are always able to access resources beneficial to their mental health [11]. Specifically, we found that CES-D scores among low-SES residents in lower-SES areas were higher than those among low-SES counterparts in higher-SES areas. This is in line with one prior study from the UK, with a more marked effect of residing in a disadvantaged neighborhood on mental health for poorer individuals [26]. And this finding may provide support for the double jeopardy hypothesis, proposing that the health of low-SES individuals will be particularly worse off if they live in lower-SES areas. This is because the negative effect of double sources of disadvantages, such as fewer individual resources and the inaccessibility of sufficient public health services and fewer community resources, on health is greater than the negative effect of only one type of socioeconomic disadvantage [47–49].

### Limitations

A significant strength of this study is that we used a longitudinal, multistage national representative population-based survey to investigate the relationship between community SES and depressive symptoms. To the best of our knowledge, our study is the first to confirm the independent association of community SES and the interaction of community- and individual-level SES with depressive symptoms among middle-aged and older adults in mainland China. Nevertheless, several limitations of this study are noteworthy. First, some variables, such as the use of antidepressants and family history of mental disorders, were not considered due to the data restrictions. Second, we cannot investigate the internal mechanisms linking community SES to depressive symptoms due to missing information on some important mediator or moderator variables in the dataset, such as social cohesion and exposure to violence. Third, despite using a longitudinal survey dataset, this study cannot obtain causal relationships.

## Conclusions

This study showed that individuals who live in lower-SES communities had more severe depressive symptoms, particularly individuals with low SES, as those residing in low-SES communities had higher depressive symptom scores than other groups. This study helps to specify the priority communities that may compensate for individual disadvantages and the priority populations, such as individuals with lower SES residing in lower-SES communities, in the prevention of depressive symptoms, providing strong empirical evidence for promoting health equity in China. Additional attention should be given to the community socioeconomic context of middle-aged and older adults with lower SES, which may be helpful to reduce SES inequalities in depressive symptoms in China. Possible interventions, such as increased community organizations, improved community-based primary health care and the development of infrastructure, should be implemented, which could increase social cohesion, develop the community social welfare, promote physical activity and access to health services and improve the overall community-level SES. Besides, prevention, screening and early diagnosis of depressive symptoms for individuals with low-SES in low-SES communities are particularly needed in consideration of their double jeopardy health risks.

## Supplementary Information

**Additional file 1.**

**Additional file 2.**

## Data Availability

The datasets analysed during the current study are available at http://charls.pku.edu.cn/en.

## Abbreviations

SES: socioeconomic status
CES-D-10: 10-item Center for Epidemiologic Studies Depression Scale
DALYs: disability-adjusted life-years
CHARLS: China Health and Retirement Longitudinal Survey
PSUs: primary sampling units
PPS: probability proportional to size
CAPIs: computer-aided personal interviews
W1: the first wave
W2: the second wave
W3: the third wave
W4: the fourth wave
ADLs: activities of daily living
HLMs: hierarchical linear regression models
ICC: interclass correlation coefficient
GHQ-30: General Health Questionnaire
PHQ-9: Patient Health Questionnaire
IDS-28: Inventory of Depression Symptomatology
