# Supplementary material for: The association between community-level socioeconomic status and depressive symptoms among middle-aged and older adults in China
Source: BMC Psychiatry. 2022 Apr 28;22:297. doi: 10.1186/s12888-022-03937-9 (PMC9047288; doi:10.1186/s12888-022-03937-9)
Supplement: Supplementary file 1 — Additional file 1. [file 12888_2022_3937_MOESM1_ESM.docx]

**Additional file 1: Table 1** Characteristics of participants from baseline CHARLS 2011.

**Table 1** Characteristics of participants from baseline CHARLS 2011

| Characteristics | Total (n=12,260) |  | Urban (n=4,821) |  | Rural (n=7,439) |
| --- | --- | --- | --- | --- | --- |
|  | N (%) or Mean (SD) |  | N (%) or Mean (SD) |  | N (%) or Mean (SD) |
| **Outcome** |  |  |  |  |  |
| CES-D-10 | 8.51(6.40) |  | 7.40(6.00) |  | 9.23(6.55) |
| **Community-level SES variables** |  |  |  |  |  |
| Average years of schooling, years | 4.71(2.22) |  | 6.16(2.39) |  | 3.78(1.49) |
| Per household income, yuan | 9489.10(8739.59) |  | 14576.68(11255.49) |  | 6191.98(4017.76) |
| Community-level SES | 0.00(1.00) |  | 0.70(1.18) |  | -0.45(0.48) |
| Community-level SES, n (%) * |  |  |  |  |  |
| Bottom tertile | 4066(33.16) |  | 554(11.49) |  | 3512(47.21) |
| Middle tertile | 4079(33.27) |  | 1078(22.36) |  | 3001(40.34) |
| Top tertile | 4115(33.56) |  | 3189(66.15) |  | 926(12.45) |
| **Individual-level SES variables** |  |  |  |  |  |
| Years of schooling, years | 4.71(4.68) |  | 6.16(4.93) |  | 3.78(4.26) |
| Per household income, yuan | 9489.10(23034.32) |  | 14576.68(33699.14) |  | 6191.98(10531.57) |
| Individual-level SES | 0.00(1.00) |  | 0.39(1.44) |  | -0.23(0.74) |
| Individual-level SES, n (%) * |  |  |  |  |  |
| Bottom tertile | 4079(33.27) |  | 963(19.98) |  | 3116(41.89) |
| Middle tertile | 4094(33.39) |  | 1459(30.26) |  | 2635(35.42) |
| Top tertile | 4087(33.34) |  | 2399(49.76) |  | 1688(22.69) |
| **Community-level sociodemographic variables** |  |  |  |  |  |
| Distance to bus stop | 2.96(9.51) |  | 1.61(9.13) |  | 3.83(9.65) |
| Days roads unpassable | 33.54(90.39) |  | 22.81(78.70) |  | 40.49(96.59) |
| Handicapped access | 1.99(1.45) |  | 2.61(1.66) |  | 1.58(1.13) |
| Employment service, n (%) |  |  |  |  |  |
| No | 9723(79.31) |  | 2641(54.78) |  | 7082(95.20) |
| Yes | 2537(20.69) |  | 2180(45.22) |  | 357(4.80) |
| Old-age income subsidies, n (%) |  |  |  |  |  |
| No | 9484(77.36) |  | 3602(74.71) |  | 5882(79.07) |
| Yes | 2776(22.64) |  | 1219(25.29) |  | 1557(20.93) |
| **Individual-level sociodemographic variables** |  |  |  |  |  |
| Age, years | 59.23(9.73) |  | 59.31(9.86) |  | 59.17(9.63) |
| Sex, n (%) |  |  |  |  |  |
| Male | 5868(47.86) |  | 2246(46.59) |  | 3622(48.69) |
| Female | 6392(52.14) |  | 2575(53.41) |  | 3817(51.31) |
| Occupation, n (%) |  |  |  |  |  |
| Agricultural work | 5814(47.42) |  | 1088(22.57) |  | 4726(63.53) |
| Nonagricultural work | 6446(52.58) |  | 3733(77.43) |  | 2713(36.47) |
| Marital status, n (%) |  |  |  |  |  |
| Unmarried | 1607(13.11) |  | 641(13.30) |  | 966(12.99) |
| Married | 10653(86.89) |  | 4180(86.70) |  | 6473(87.01) |
| ADLs, n (%) |  |  |  |  |  |
| No-impaired | 10130(82.63) |  | 4183(86.77) |  | 5947(79.94) |
| Impaired | 2130(17.37) |  | 638(13.23) |  | 1492(20.06) |

*Note:* CES-D-10 = 10-item short form of the Center for Epidemiologic Studies Depression scale; SES = socioeconomic status; ADLs = activities of daily living.

*Variable was treated as a continuous variable but is presented categorically for descriptive purposes.
