# Supplementary material for: The association between community-level socioeconomic status and depressive symptoms among middle-aged and older adults in China
Source: BMC Psychiatry. 2022 Apr 28;22:297. doi: 10.1186/s12888-022-03937-9 (PMC9047288; doi:10.1186/s12888-022-03937-9)
Supplement: Supplementary file 2 — Additional file 2. [file 12888_2022_3937_MOESM2_ESM.docx]

**Additional file 2:** Tests for autocorrelation, homoscedasticity, normality and multicollinearity of multilevel modeling.

**
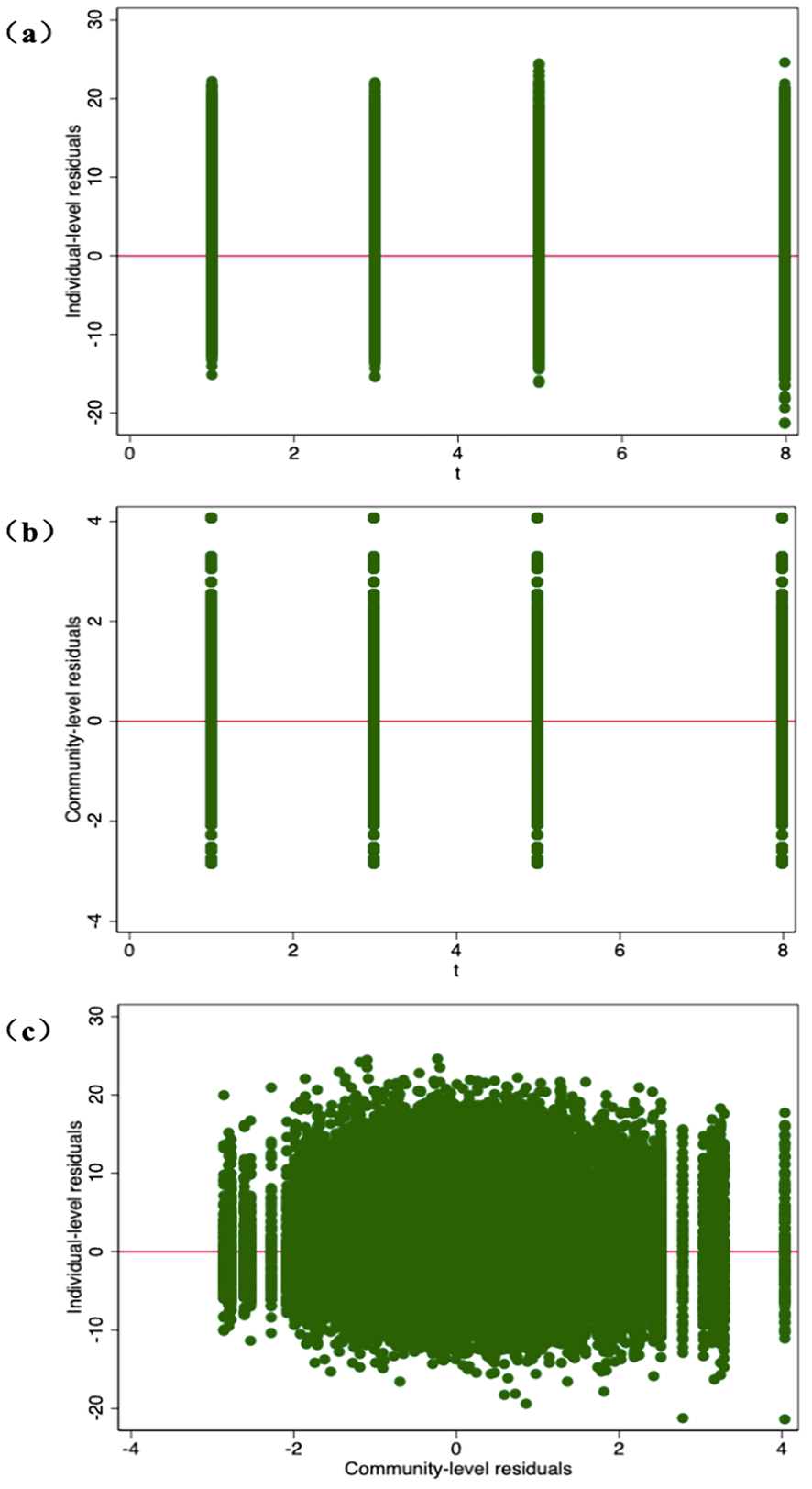
**

**Fig. 1** Tests for autocorrelation of multilevel modeling. (a) tested the independence between individual-level residuals and time; (b) tested the independence between community-level residuals and time; (c) tested the independence between individual-level residuals and community-level residuals.


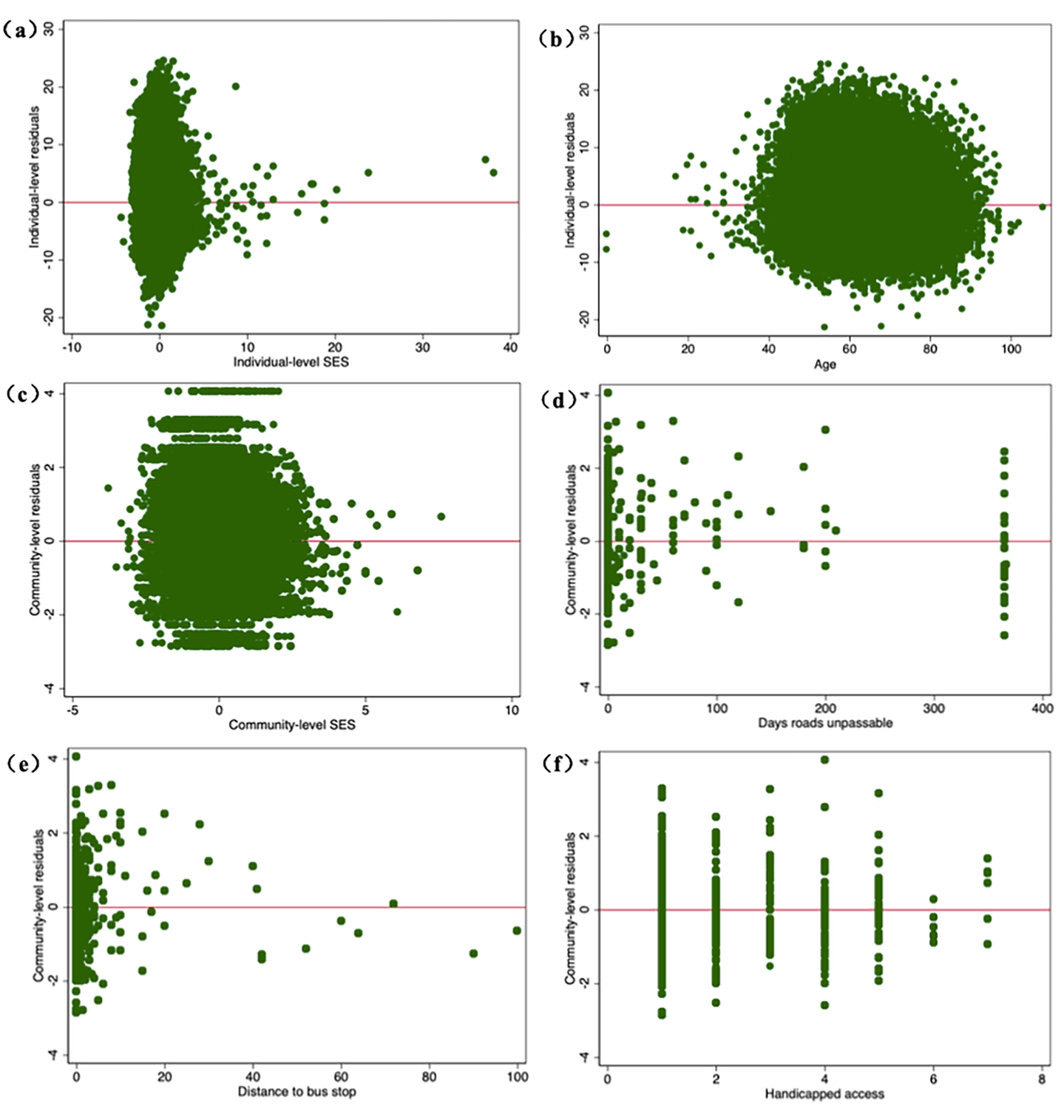


**Fig. 2** Tests for homoscedasticity of multilevel modeling. (a) and (b) presented the distributions of residuals in individual-level SES and age at the individual level, respectively; (c), (d), (e) and (f) presented the distributions of residuals in community-level SES, days roads unpassable, distance to bus stop, and handicapped access at the community level, respectively.


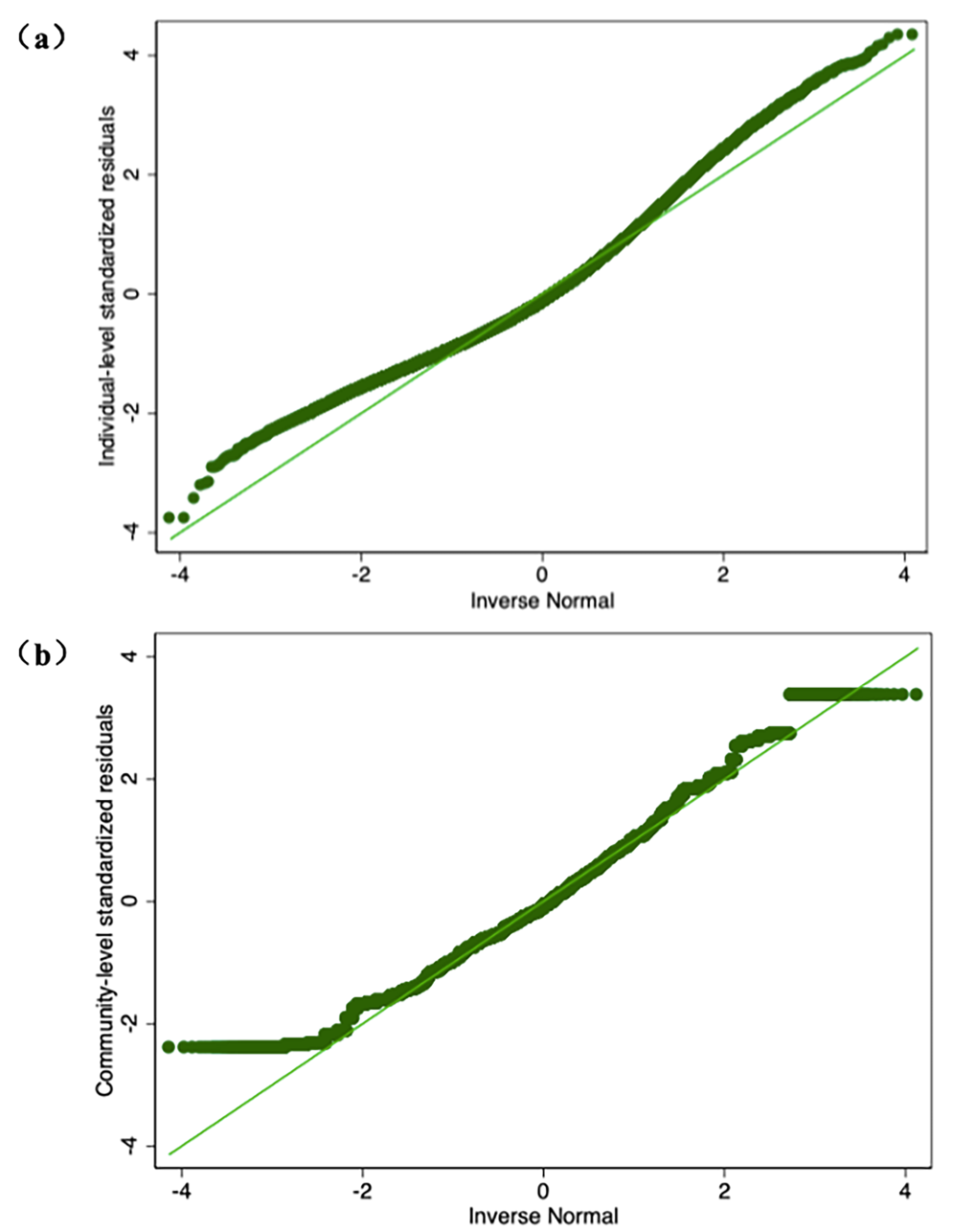


**Fig. 3** Tests for normality of multilevel modeling. (a) and (b) showed the normal quantile-quantile plots (Q-Q plots) at the individual level and the community level.

**Table 1** Tests for multicollinearity of multilevel modeling (VIF values).

| Variable | Model 1 | Model 2 | Model 3 |
| --- | --- | --- | --- |
| Community-level SES | 1.62 | 1.96 | 1.97 |
| Individual-level SES |  | 1.54 | 1.86 |
| Community-level SES x Individual-level SES |  |  | 1.31 |
| Distance to bus stop | 1.04 | 1.04 | 1.05 |
| Days roads unpassable | 1.05 | 1.05 | 1.05 |
| Handicapped access | 1.21 | 1.21 | 1.21 |
| Employment service | 1.39 | 1.39 | 1.40 |
| Old-age income subsidies | 1.01 | 1.01 | 1.01 |
| Age | 1.20 | 1.27 | 1.29 |
| Sex | 1.03 | 1.09 | 1.10 |
| Occupation | 1.28 | 1.29 | 1.67 |
| Marital status | 1.15 | 1.15 | 1.29 |
| ADLs | 1.08 | 1.08 | 1.15 |
| Mean VIF | 1.23 | 1.29 | 1.32 |

*Note:* VIF = Variance Inflation Factor.

Model 1: adjusted for community-level sociodemographic variables (distance to bus stop, days roads unpassable, handicapped access, employment service, old-age income subsidies) and individual-level sociodemographic variables (age, sex, residence, occupation, marital status, ADLs).

Model 2: adjusted for Model 1 criteria and individual-level SES.

Model 3: adjusted for Model 2 criteria and the interaction between community-level and individual-level SES.
